# Seasonally Dependent Relationships between Indicators of Malaria Transmission and Disease Provided by Mathematical Model Simulations

**DOI:** 10.1371/journal.pcbi.1003812

**Published:** 2014-09-04

**Authors:** Erin M. Stuckey, Thomas Smith, Nakul Chitnis

**Affiliations:** 1 Swiss Tropical and Public Health Institute, Basel, Switzerland; 2 University of Basel, Basel, Switzerland; Imperial College London, United Kingdom

## Abstract

Evaluating the effectiveness of malaria control interventions on the basis of their impact on transmission as well as impact on morbidity and mortality is becoming increasingly important as countries consider pre-elimination and elimination as well as disease control. Data on prevalence and transmission are traditionally obtained through resource-intensive epidemiological and entomological surveys that become difficult as transmission decreases. This work employs mathematical modeling to examine the relationships between malaria indicators allowing more easily measured data, such as routine health systems data on case incidence, to be translated into measures of transmission and other malaria indicators. Simulations of scenarios with different levels of malaria transmission, patterns of seasonality and access to treatment were run with an ensemble of models of malaria epidemiology and within-host dynamics, as part of the OpenMalaria modeling platform. For a given seasonality profile, regression analysis mapped simulation results of malaria indicators, such as annual average entomological inoculation rate, prevalence, incidence of uncomplicated and severe episodes, and mortality, to an expected range of values of any of the other indicators. Results were validated by comparing simulated relationships between indicators with previously published data on these same indicators as observed in malaria endemic areas. These results allow for direct comparisons of malaria transmission intensity estimates made using data collected with different methods on different indicators. They also address key concerns with traditional methods of quantifying transmission in areas of differing transmission intensity and sparse data. Although seasonality of transmission is often ignored in data compilations, the models suggest it can be critically important in determining the relationship between transmission and disease. Application of these models could help public health officials detect changes of disease dynamics in a population and plan and assess the impact of malaria control interventions.

## Author Summary

While malaria is still a major public health problem in many parts of the world, control programs have greatly reduced the burden of disease in recent years and many countries are now considering the goal of elimination. Unfortunately, malaria transmission becomes more difficult to measure when it is low because traditional methods involve capturing mosquitoes; an expensive and time-consuming technique. To measure transmission in areas without adequate field data, we run simulations of a mathematical model of malaria over a range of transmission intensities and seasonal patterns to examine how different measurements of malaria (prevalence, clinical disease, and death) relate to each other, how they relate to transmission, and if the relationships are likely to vary by seasonal pattern of transmission. These simulated relationships allow us to translate easily measured data, such as clinical case incidence seen at health facilities, into estimates of transmission. This technique can help public health officials plan and assess the impact of malaria control interventions, even in areas without intensive research activities.

## Introduction

Evaluating the effectiveness of malaria control interventions on the basis of their impact on transmission is increasingly important as countries consider elimination as well as malaria control. However, direct measurement of transmission, such as by the entomological inoculation rate (EIR) (a measure of human exposure defined by the number of infective mosquito bites per human in a given time period), involves mosquito capture. This is extremely labor-intensive, and is only reliable in high transmission areas and seasons [1]. In areas of low transmission, or during dry seasons, identifying a sufficient number of sporozoite-positive mosquitoes makes this exercise excessively time- and resource-intensive, often precluding collection of a full year's worth of data and making estimates of seasonality challenging. Alternatives are to estimate transmission rates from sero-conversion rates [2], [3] or by calculating force of infection (FOI) from combining information on prevalence and treatment [4]. Estimating both the exposure to infectious mosquitoes and subsequent FOI from parasite prevalence in areas of high transmission is difficult due to superinfection and immunity. Mathematical models are useful in examining relationships between malaria indicators, allowing translation of routine health center data into measures of transmission and addressing concerns with previously implemented methods of measuring transmission [5].

Understanding the seasonal pattern of malaria transmission is important for planning control interventions, for example the timing of deploying indoor residual spraying (IRS) and seasonal malaria chemoprophylaxis (SMC) which are implemented ahead of the peak transmission months. Given the wide range of seasonal patterns combined with transmission intensities that exist in areas of the world with malaria transmission, and due in large part to the absence of robust field data, the effect of seasonality on the relationship between malaria indicators has not been studied in great detail. Attempts have been made to define [6], [7] and quantify [8] the relationship between seasonally varying covariates and transmission based on available studies on malaria transmission and disease burden, but results for the latter were only found to be reliable in areas of very high transmission (EIR>100 infectious bites per person per year) [6].

One approach for quantifying transmission in areas without EIR data is to use simulation models to analyze how different malaria indicators (parasite prevalence, prevalence of uncomplicated and severe episodes, mortality) relate to each other, and how they relate to transmission as measured by EIR [5]. To validate such models, a straightforward approach would be to compare the simulated relationships between indicators to those observed in the field. However, when relationships between indicators differ in places with disparate patterns of seasonality, such an approach becomes challenging. This study uses simulation models to analyze whether relationships between malaria indicators are likely to vary by intensity and pattern of seasonality. Analysis of these simulation results can help identify the best way of quantifying transmission for the purposes of specifying the seasonal patterns to drive existing models of *Plasmodium falciparum* dynamics. This in turn will assist in planning for malaria control by allowing for the selection of interventions tailored to the level of transmission in a given location, and monitoring the effectiveness of those interventions by their impact on transmission.

## Methods

### OpenMalaria transmission model simulation

This experiment utilizes an ensemble of simulation models of transmission of malaria developed by a team at the Swiss Tropical and Public Health Institute (Swiss TPH) and Liverpool School of Tropical Medicine. These models form part of the OpenMalaria platform that makes the considerable code base written in C++ accessible to the public through an online wiki [9]. Based on a stochastic series of parasite densities for individual infections, stochastic individual-based models of malaria in humans [10]–[12] are linked to a periodically-forced model of malaria in mosquitoes [13] in order to simulate the dynamics of malaria transmission and the impact of intervention strategies for malaria control. Details of the methods to create and parameterize the transmission model used in this project have been previously published [10]–[13] and therefore are not covered in this paper. Models are fitted to 10 objectives using 61 standard scenarios as described in Smith et al. 2008 [11]. The transmission model is calibrated by the seasonal pattern of the EIR with units of infectious bites per person per year. Simulations were run for one human life span to induce a stable level of immunity in the population. Each simulation was repeated on an ensemble of 14 model variants with varying assumptions on mass action, heterogeneity of exposure, decay of acquired immunity, co-morbidities, and access to treatment as described in Smith et al [12] to address model uncertainty, with five random seeds to address stochasticity.

### Study design

The overall objective of estimating transmission in areas without EIR data was addressed by applying the OpenMalaria modeling platform to simulate malaria with different levels of transmission and patterns of seasonality observed in malaria-affected locations, and deriving outputs for all other malaria indicators. **Table 1** describes the indicators chosen as simulation outputs that were evaluated in this study. Relationships between all indicators for the different values of EIR and different seasonality profiles were estimated from simulation results (described below) using Stata v12 (College Station, TX). For this process the indicators were calculated for the whole population, with the exception of the relationships involving mortality which were limited to children under five due to a lack of data in older age groups for validation purposes.

**Table 1:** Malaria indicators described in this study and their definitions for the purposes of this study.

Indicator name	Definition	Transformation*
Entomological Inoculation Rate (EIR)	Annual average number of infectious bites received from a malaria vector per person	Logarithmic
Parasite prevalence	Proportion of the population (all ages) with detectable parasitaemia (greater than 100 parasites per microliter)	Logit
Uncomplicated episodes	Annual average number of uncomplicated clinical episodes of malaria per person (all ages)	Logarithmic
Severe episodes	Annual average number of severe clinical episodes of malaria per 1,000 people (all ages)	Logarithmic
Mortality	Annual average number of deaths due to malaria in children under 5 per 1,000 people	Logarithmic

* Transformation used in fractional polynomial analysis.

### Scenario design

The baseline scenario used in these experiments was based on a scenario previously parameterized for the Rachuonyo South district in the highlands of western Kenya [14]. The model assumes no interventions beyond case management through the health system as described in Tediosi et al. [15], a main vector of *A. gambiae* s.s., and artemisinin combination therapy (ACTs) as the first line antimalarial. Simulations were run on a population of 100,000 individuals over three years with monthly surveys of malaria outcomes.

### Seasonality index

To quantify the “amount” of seasonality in a location a seasonality index (*φ*) was defined in order to describe the variations in transmission within one year in a given location. The methodology presented here is general and can be used for any measure of transmission, but the example below is used with EIR.

We let *T* denote the period (1 year) and let *f (t)* be a positive continuous periodic function that denotes transmission at time *t*, with *f* (*t*+*T*) = *f* (*t*)>0 for all *t*≥0. The mean level of transmission (over 1 year) is,
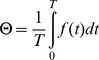
In a similar manner to the coefficient of variation in statistics, we define *φ* as the normalized square root of the integral of the squared difference between *f(t)* and its mean,
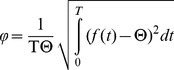
This seasonality index, *φ*, allows us to quantify the level of seasonality of transmission in a given location with one positive real number, differentiating between “amounts” of seasonality for transmission patterns with the same number of peaks. Because malarious areas in general have either one or two peak transmission seasons, there could be seasonality patterns in different locations that lead to the same seasonality index, 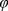. We therefore label the seasonality profile with both the seasonality index and the number of peaks.

### Seasonality profiles

**Figure 1:**
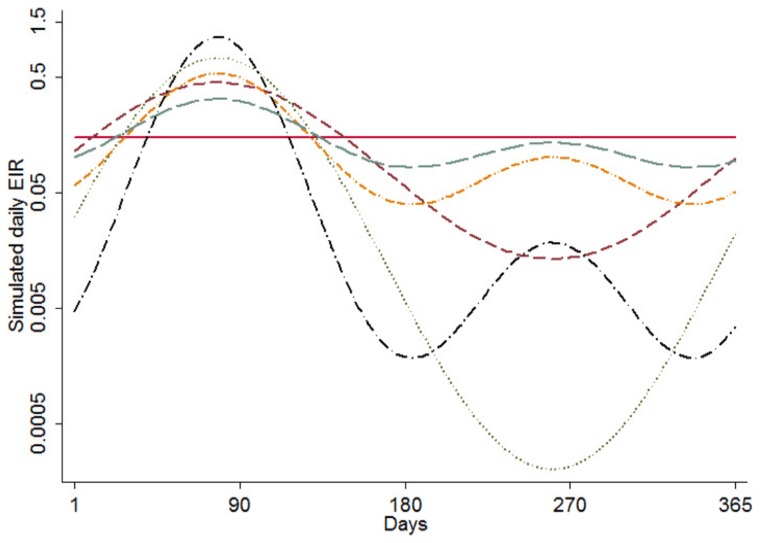
Annual pattern of transmission, defined as the simulated daily EIR, for each seasonality profile as described by (the seasonality index *φ*, number of peaks). Unbroken red line represents (0, 0). Brown dashed line represents (1,1). Orange dotted-dashed line represents (1,2). Green dotted line represents (2,1). Black dotted-dashed line represents (2,2). Blue dashed line represents (0.5, 2).

The simulations described here treat transmission in the absence of interventions as periodic with a one year period [13]. One scenario with a seasonality pattern of constant annual transmission (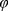 = 0) and five scenarios with varying seasonal transmission patterns (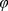 = 1, one peak; 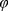 = 1, two peaks; 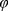 = 0.5, two peaks; 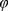 = 2, one peak; 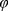 = 2, two peaks) were created, described in **Table 2** and **Figure 1**.

**Table 2:** Seasonality patterns of transmission observed malaria-endemic areas.

Seasonality pattern ID	Seasonality index ( 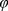 )	Number of peaks	Description
**0, 0**	0	0	No seasonality – constant transmission throughout the year
**1, 1**	1	1	Medium seasonality, one transmission season
**1, 2**	1	2	Medium seasonality, two transmission seasons
**0.5, 2**	0.5	2	Low seasonality, two transmission seasons
**2, 1**	2	1	High seasonality, one transmission season
**2, 2**	2	2	High seasonality, two transmission seasons

These six patterns were chosen to represent the range of seasonal patterns of malaria transmission existing in the malaria endemic world, namely because there are usually not more than two peak transmission seasons. The seasonality profiles with 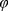 = 2 exhibit large variations in seasonality. For 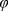 = 2 with one peak, 86% of annual transmission is focused in the three peak transmission months, while for 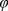 = 2 with two peaks, the peak is narrower with 95% of annual transmission occurring in the three months of the higher peak. The results of what this means for prevalence and morbidity over one year can be found in **Figure S1 in Text S1**. Seasonality patterns were repeated for eleven values of annual average EIR from 0.5 to 365. Complete details of the methods behind the experiment creation can be found in **Text S1**. The relationships between malaria indicators were estimated using fractional polynomial regression as described in more detail in **Text S2**.

### Model validation

In order to gauge the model's ability to reproduce field data, a validation exercise was completed by comparing simulation results to data not used in the original process of model fitting from previously published studies. The relationships for validation, the datasets used and how they relate to model fitting are described in **Table S1**.

While the annual average EIR in the scenarios used for estimating the relationships between malaria indicators were capped at a value of 81.4, scenarios for validation were simulated up to an average of 365 infectious bites per person per year. This tailors the analysis to low- to mid-range values of annual average EIR where this tool will be the most applicable, while still allowing for a more comprehensive range of annual average EIRs that appear in the validation datasets.

## Results

### Indicators as a function of entomological inoculation rate (EIR)

**Figure 2:**
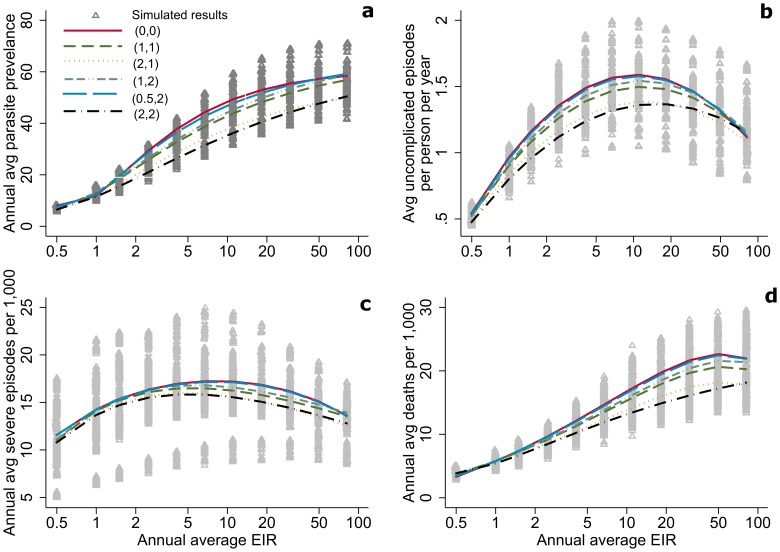
Relationship of parasite prevalence (a), uncomplicated episodes (b), severe episodes (c), and mortality (d) to annual average EIR by seasonality index (*φ*). Triangles represent simulated results. The lines show the estimated relationship between indicators from the simulation runs, fitted using fractional polynomial regression, for each pattern of seasonality as described by (the seasonality index 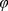, number of peaks) (**Figure 1**). Unbroken red line represents (0, 0). Brown dashed line represents (1,1). Orange dotted-dashed line represents (1,2). Green dotted line represents (2,1). Black dotted-dashed line represents (2,2). Blue dashed line represents (0.5, 2).

When analyzing the relationship between EIR and other malaria indicators, the differences between seasonality profiles are greatest at moderate levels of EIR (**Figure 2a–d**). Results are similar between seasonality profiles at both ends of the EIR spectrum for uncomplicated and severe disease, but seasonality impacts the relationship with prevalence and mortality more at higher values of EIR (**Figure 2a–d**).

**Figure 3:**
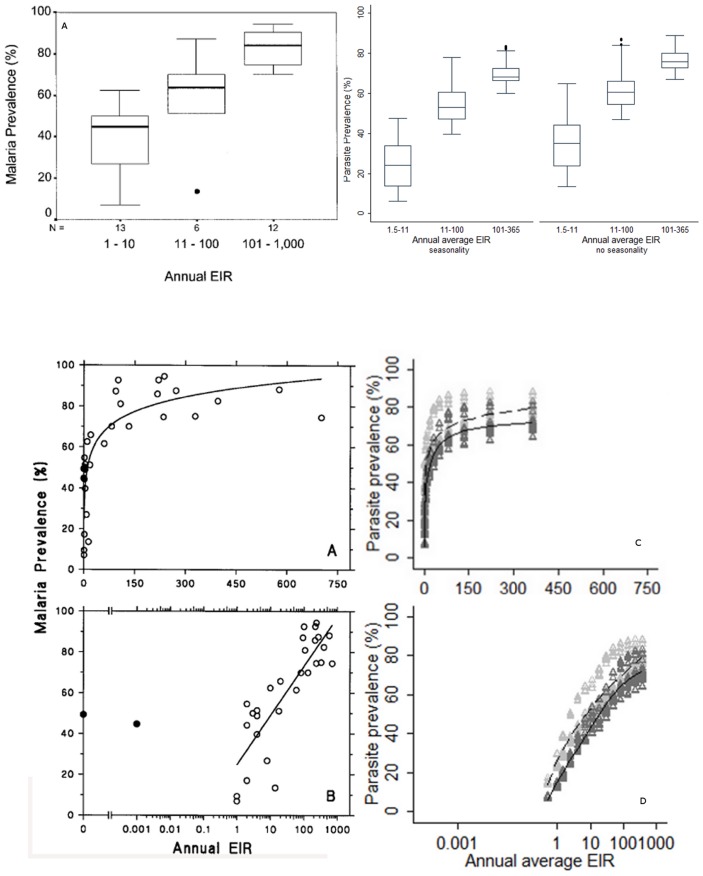
The relationship between prevalence (defined as the maximum recorded parasite prevalence rate in any given age group) and EIR from Beier et. al [17] (3.1 a, 3.2 a–b) and OpenMalaria simulations (3.1 b, 3,2 d). In **3.1** the mean value is shown as a line inside the box, the 25th to 75th percentile is shown by the box, and the range of values is shown by the lines outside the box. In **3.2** grey triangles represent simulation results without (light gray) and with (dark gray) seasonality as described by (the seasonality index 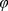, number of peaks) (**Figure 1**). The lines show the estimated relationships with seasonality (2, 2) (dashed) and without seasonality (0, 0) (unbroken) using fractional polynomial regression. Figures **3.1a** and **3.2a–b** have been reproduced from Beier et al [17] with permission.

The Beier et al. dataset, describing the relationship between EIR and parasite prevalence in children under five in sites across Africa, has been applied for a previous validation of the OpenMalaria model [16]. One site out of 31 as published separately was used to fit the model for incidence of asexual blood stage infection, as indicated in **Table S1**. Compared to the results presented in Beier et al. [17], simulation results are within the range of observed values for low and medium values of EIR, but predict a slightly lower prevalence in extremely high EIR settings, especially in a setting with no seasonality (**Figure 3**). Perhaps this is because observed results reach up to 1,000 infectious bites per person per year while the simulated scenarios were capped at 365. While the observed relationship is fitted as log-linear, the simulated relationship starts levelling off at an EIR of 100.

### Indicators as a function of parasite prevalence

**Figure 4:**
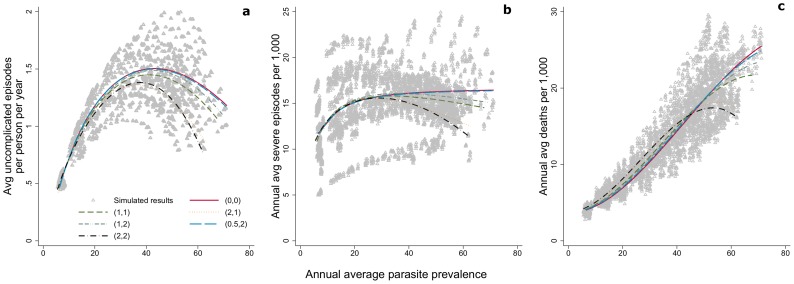
Relationship of uncomplicated episodes (a), severe episodes (b), and mortality (c) to parasite prevalence by seasonality index. Triangles represent simulated results. The lines show the estimated relationship between indicators from the simulation runs, fitted using fractional polynomial regression, for each pattern of seasonality as described by (the seasonality index 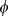, number of peaks) (**Figure 1**). Unbroken red line represents (0, 0). Brown dashed line represents (1,1). Orange dotted-dashed line represents (1,2). Green dotted line represents (2,1). Black dotted-dashed line represents (2,2). Blue dashed line represents (0.5, 2).

The relationship between parasite prevalence and uncomplicated episodes is non-monotonic (**Figure 4a**) for all values of 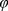. It can be noted that the simulated relationship between parasite prevalence and severe disease shows more stochasticity than the other relationships with parasite prevalence in areas of lower prevalence (**Figure 4b**). This variation can be attributed to model uncertainty, in particular differing assumptions about access to treatment, rather than to the effect of seasonality. For uncomplicated disease, severe disease and mortality, the effect of seasonality is greater in areas of higher parasite prevalence; the variation increases once prevalence reaches 40% (**Figure 4a–c**).

**Figure 5:**
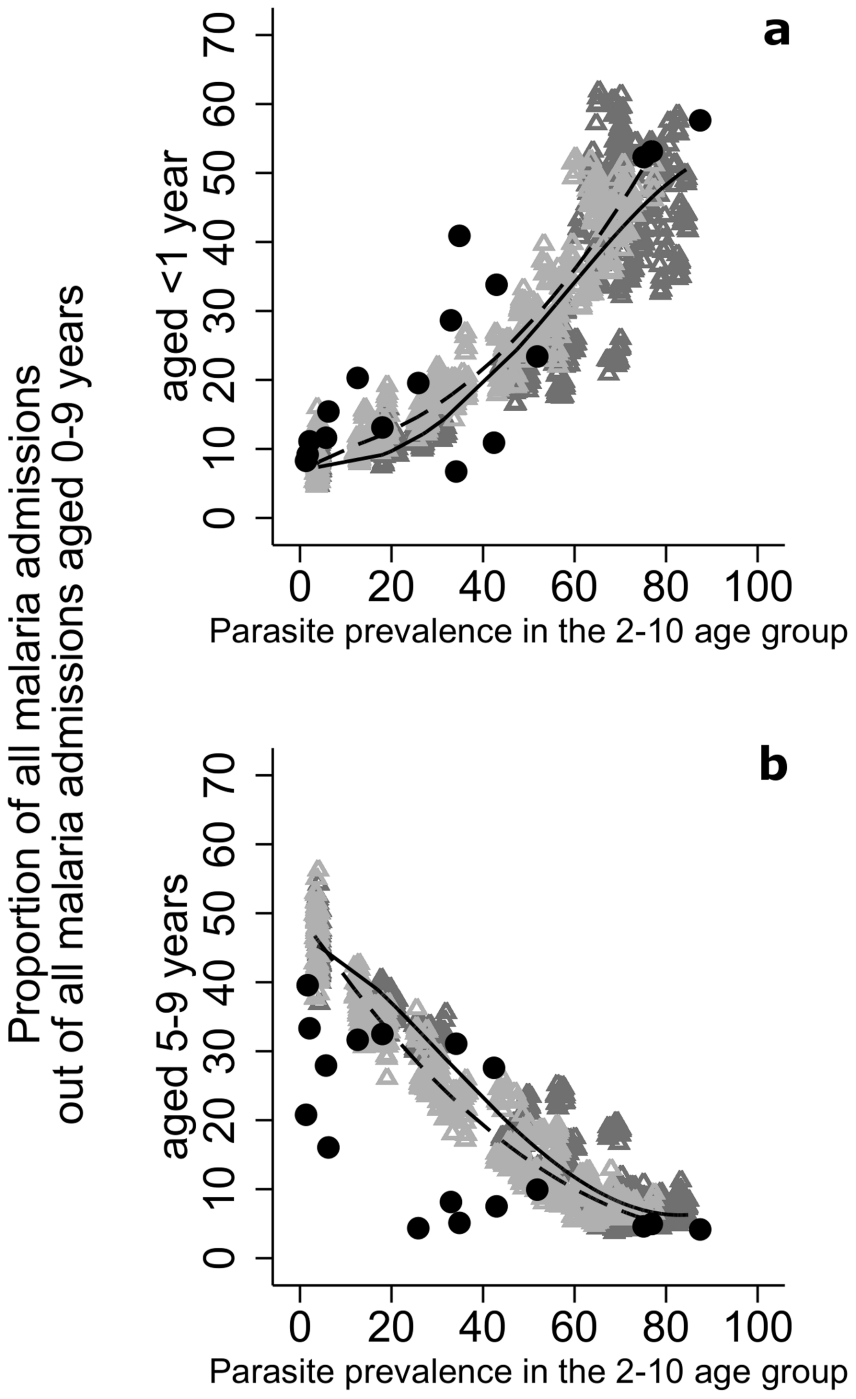
Relationship between the proportion of paediatric severe malaria in children under 1 year (a) and children aged 5–9 years (b) and parasite prevalence in the 2–10 age group from Okiro et. al, [18] (black circles) and OpenMalaria simulations (grey triangles). Triangles represent simulation results with (dark gray) and without (light gray) seasonality. Lines show the estimated relationships with (dashed) and without (unbroken) seasonality using fractional polynomial regression.

Compared to the results presented in Okiro et al. [18] the model is able to reproduce the general pattern of the relationship between severe pediatric malaria and prevalence in children aged 2–10 in children under 1 year as well as in children aged 5–9, with the burden of malaria moving to older age groups as prevalence is reduced (**Figure 5**).

**Figure 6:**
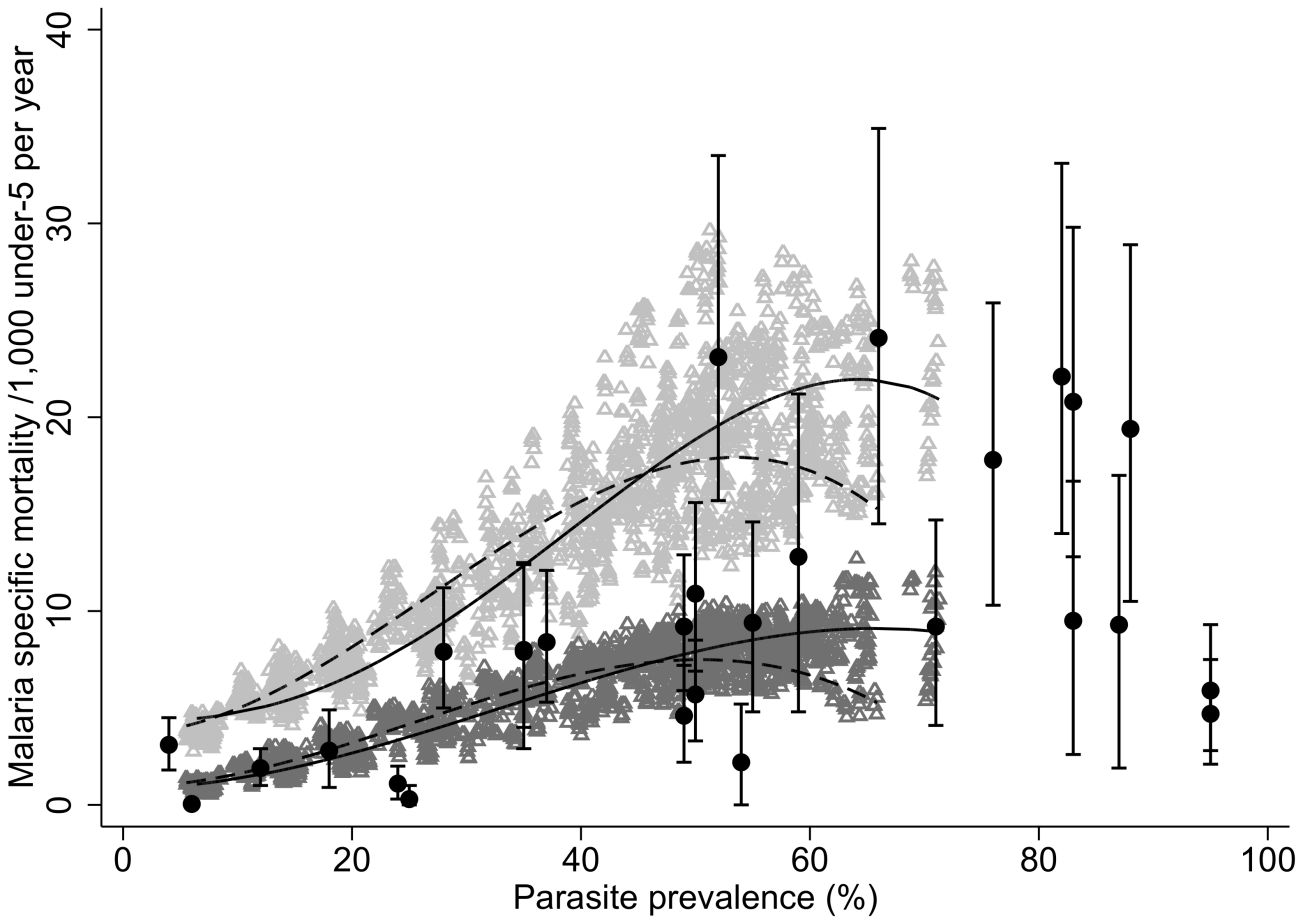
Relationship between mortality in children under 5 and average all-age parasite prevalence as described in Korenromp et. al [19] (black circles) and OpenMalaria simulations (triangles) for all deaths (light gray) and direct deaths only (dark gray). Lines show the simulation-based estimated relationships with seasonality (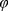 = 2, 2 peaks) (dashed) and without seasonality (φ = 0, 0 peaks) (unbroken) using fractional polynomial regression. The observed values from Korenromp et. al are results of verbal autopsy which do not specify direct malaria deaths as opposed to indirect malaria deaths.

Compared to the results presented in Korenromp et al. [19], which describes the relationship between parasite prevalence and both malaria-specific and all-cause mortality in children under 5, the model is able to capture the general pattern for the relationship between malaria-specific mortality in children under five for low and moderate prevalence settings (**Figure 6**). There appears to be variation across sites in the observed data that may be explained by the ability of verbal autopsy to capture indirect deaths due to malaria in different settings [20]. Nine sites (for which EIR estimates were available) out of the 28 sites included in the study were used to fit the model of direct malaria mortality in relation to EIR, as indicated in **Table S1**.

### Indicators as a function of uncomplicated episodes

**Figure 7:**
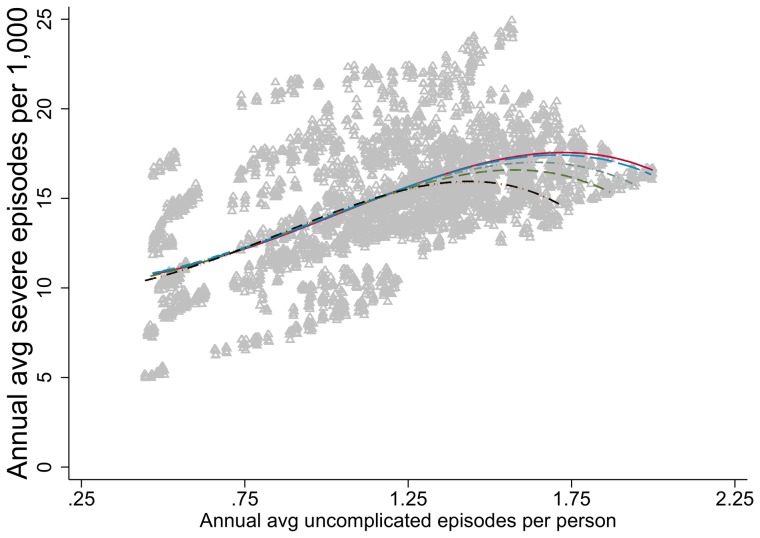
Relationship of severe episodes to uncomplicated episodes by seasonality index. Triangles represent simulated results. The lines show the estimated relationship between indicators from the simulation runs, fitted using fractional polynomial regression, for each pattern of seasonality as described by (the seasonality index 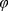, number of peaks) (**Figure 1**). Unbroken red line represents (0, 0). Brown dashed line represents (1,1). Orange dotted-dashed line represents (1,2). Green dotted line represents (2,1). Black dotted-dashed line represents (2,2). Blue dashed line represents (0.5, 2).

At lower numbers of uncomplicated episodes per person per year, seasonality does not play a role in the relationship with severe episodes (**Figure 7**). The curves separate at levels above 1.25 uncomplicated episodes per person per year with two-peak scenarios 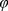 = 1 and 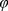 = 2 diverging from the other values of 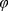 (**Figure 7**). The scatter plot of simulation results showed no discernible relationship between mortality and either uncomplicated or severe episodes, and are therefore not shown here.

### Age prevalence curves by indicator

Age prevalence curves are validated by comparing simulation results to those presented in Carneiro et al, which report on the age distribution of children with clinical malaria, hospital admissions with malaria and malaria-diagnosed mortality for different categories of intensity and seasonality of malaria transmission identified from a systematic review epidemiological studies [6].

It should be noted that there are differences in the classification of degree of seasonality between the observed and simulated data. Carneiro and colleagues describe settings with marked seasonality as those with greater than or equal to 75% of episodes concentrated less than or equal to 6 months of the year. In the OpenMalaria simulations, marked seasonality is defined as the setting with 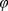 = 2.

**Figure 8:**
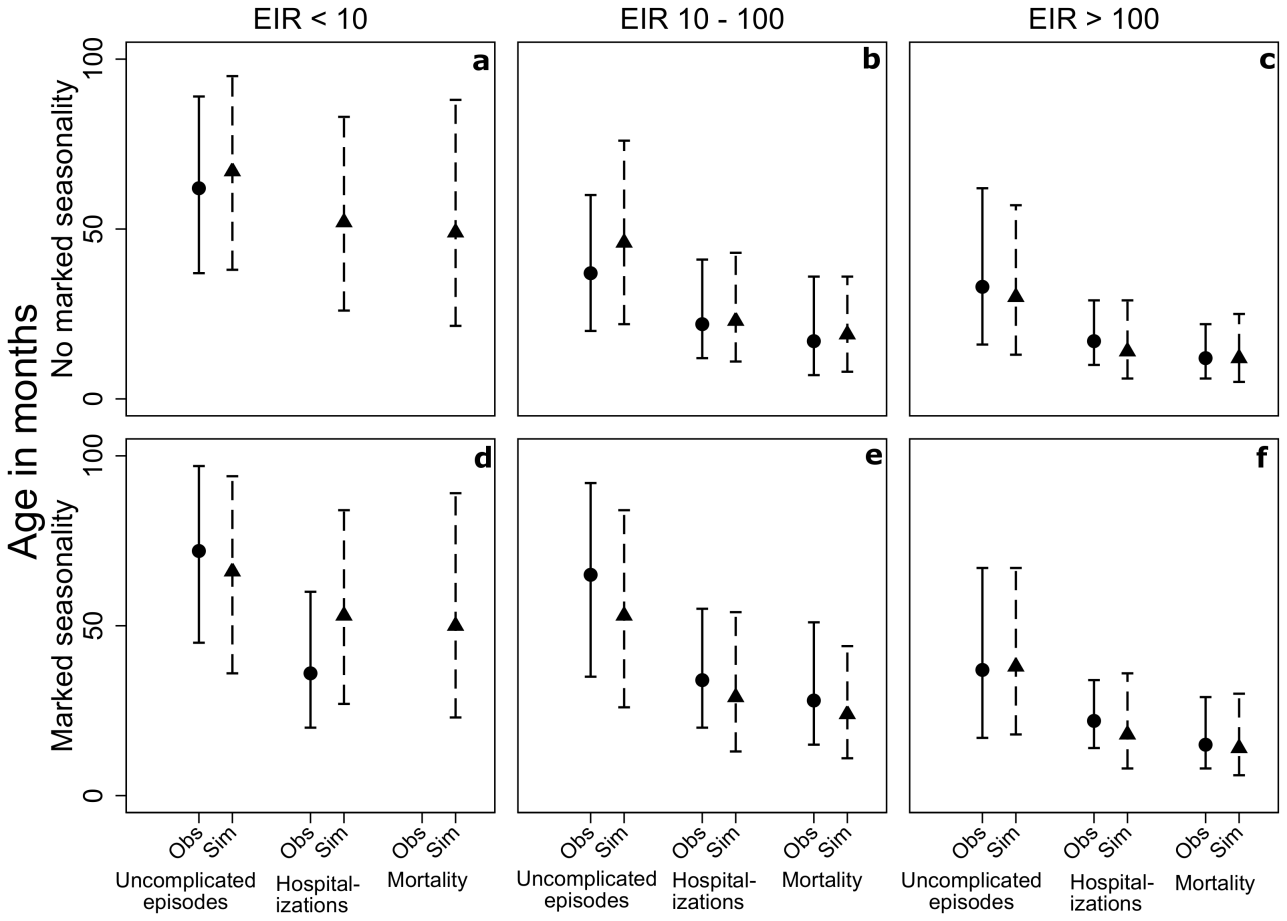
Median ages and inter-quartile range age prevalence curves in months of age by annual average EIR levels of <10 (a, d), 10–100 (b, e), and >100 (c, f), and seasonality patterns *φ* = 2, 2 peaks (a–c) and *φ* = 0, 0 peaks (d–f) for uncomplicated episodes, malaria hospitalizations, and mortality as observed in Carneiro et al. [6] (circles, unbroken lines) and simulated by the OpenMalaria model (triangles, dashed lines).

The reported estimated median ages and inter-quartile ranges (defined as the 50th percentile of the best-fitting distribution for each outcome and transmission scenario) from these fitted models for each level of transmission and level of seasonality are compared to estimates from fitted OpenMalaria simulation results to validate age prevalence curves of the malaria indicators mentioned above. In all cases, the results of the OpenMalaria simulations are comparable to the previously published results (**Figure 8**).

## Discussion

Due to the lack of understanding of the relationship between EIR and other malaria indicators based on challenges in measuring EIR from entomological studies, modeling is able to further define the relationships between indicators and help clarify details of what cannot measured from field studies but is nonetheless necessary knowledge about malaria indicators. This is of value for malaria control program managers because it provides insight on transmission without substantial field studies. These models can be used to simulate the likely range of values in areas without access to adequate field data.

Empirical studies of the relationships between different malaria indicators are challenging because these relationships may in principle be affected by many, often poorly characterized, contextual factors, with the degree of seasonality being possibly one of the most important. The original fitting of the OpenMalaria model parameters to multiple field datasets used a standard pattern of seasonality of transmission from Namawala, Tanzania; effects of seasonality observed in these results are thus not an artifact of the fitting process. Simulations suggest that with equal levels of average annual transmission, the level of seasonality, i.e. whether malaria transmission is fairly constant over the course of a year versus peaks in certain months, affects the relationship between malaria indicators. An increase in the degree of seasonality has a greater impact on outcomes with moderate levels of EIR and prevalence. There is greater stochasticity in simulation results for scenarios with higher amplitude of the annual cycle compared to scenarios with a constant level of transmission.

There have been previous attempts to create a measure for the seasonality of malaria transmission [21]–[23], mainly relying only on rainfall and/or vector abundance to describe the proportion of transmission occurring within a certain number of months. The approach to developing the seasonality index presented here is in response to the need to provide a quantitative metric for differences between seasonal patterns. Results indicate that this index does not distinguish well between patterns that have a different number of peaks (**Figure 2**); therefore the number of peaks should also be noted in any analysis of studies that employ this index. Areas with seasonal malaria transmission typically have substantial variation in rainfall and transmission with numerous small peaks, but normally only have one or two main seasons. The total number of peaks can thus be assumed to be limited to a maximum of two.

The difference in results for different patterns within the same seasonality index calls into question the assumptions behind the drivers of the relationships between malaria indicators. Scenarios with a higher degree of seasonality, regardless of number of peaks, return lower levels of prevalence, disease and mortality for a given level of transmission. An important driver is multiple concomitant events; when two illness episodes occur at the same time they are only considered as one, which may occur more frequently in high seasonality scenarios. At more mild patterns of seasonality, this phenomenon is only seen at higher levels of transmission. These results also potentially indicate an effect on acquisition of immunity in these settings, a consideration when modeling the relationship between transmission and the acquisition of immunity in a population. Several model variants differ in their assumptions about immunity [12], and while outside the scope of this paper, an important question for future investigation would be the impact of this aspect of the models variants and the effect, if any, that occurs for different seasonal patterns of transmission.

Results indicating the impact of seasonality on the relationship between malaria indicators is relevant to malaria epidemiology and control because, as has been described in Carneiro et al [6], areas with similarly high average annual prevalence result in less frequent cases of malaria in highly seasonal settings. A focused empirical analysis of this effect would be another welcome addition to the understanding of the subject.

Access to treatment has the potential to impact the relationships between transmission and other malariological indicators such as severe disease and mortality. The higher the proportion of malaria cases that are treated with effective antimalarials the more the parasite reservoir in the human host population is suppressed, the fewer gametocytes are available, and the less likely it is that mosquitoes are infected. The authors are not aware of any empirical studies of the relationship between access to treatment and population-level health outcomes. However, recent work by Briët and Penny investigates the impact of access to treatment on the OpenMalaria model [24]. The relationships between severe episodes and other indicators (**Figures 2c**, **4b** **,** **7**) may depend more on access to effective case management, indicated by the variance in simulation results which is due to model uncertainty rather than the effect of seasonality.

There are direct implications on control programs for the relationship between seasonality and the expected number of uncomplicated cases for a given level of parasite prevalence. Locations with poor monitoring and surveillance systems resulting from complex emergencies or insufficient reach of the public sector may have readily-available parasite prevalence data as a result of research activities. These results may impact how routine data from the case management system in these locations are able to be used to inform study design for the implementation of seasonality-dependent interventions such as IRS and SMC.

Two sources mentioned in this model validation were also used in the original model fitting [12]. However, as indicated in the Results section and in **Table S1**, the relationships used here for validation were not the same relationships (Korenromp et al.) or subsets of data (Beier et al.) used for fitting. Although both help parameterise the model, because this process was independent to the relationships being validated, they can therefore be treated as available for validation.

Each simulation result is a point in multidimensional space with each dimension corresponding to one malaria indicator. However, to determine the relationship between any two indicators, all simulation points are projected onto a two-dimensional space where the relationship is estimated through fractional polynomial regression. Due to this projection, when two indicators have a monotonically-increasing relationship with a third indicator, they may not necessarily have a monotonically-increasing relationship with each other. For example, while simulated parasite prevalence and mortality both increase with increasing annual average EIR, the same effect will not necessarily be seen on mortality in conditions of increasing prevalence. Similarly, the effects of seasonality appear to decrease as EIR increases, but increase as prevalence increases.

While the range of transmission levels and patterns represented in this study are designed to cover a large proportion of malaria endemic areas, there are areas with contexts that will fall outside the scope of this work. There remain areas with extremely high transmission beyond an annual average EIR of 81.4 at which this analysis is capped, but these programs are unlikely to be at a stage of malaria control to benefit from applying the methods described in this paper for fine-tuning malaria control interventions as vector control interventions can be effectively utilized to substantially reduce malaria transmission to moderate levels and transmission can be adequately measured with entomological methods.

Simulated results were limited to annual average EIR values greater than 0.5. In very low transmission settings infections are sporadic and could be better captured with epidemic models. At very low annual average transmission rates malaria can be sustained by regular importation or the presence of hotspots. The relationships between malaria indicators then depend critically on the degree of transmission heterogeneity and interactions between sub-populations. In these settings, estimating transmission through using serology to estimate EIR or force of infection may be more suitable. Although not currently available in the OpenMalaria transmission model, force of infection and serology will be important components to add to future versions to better simulate the current practice of measuring transmission at low values of EIR. With the inclusion of these indicators, the new model can be calibrated with data on incidence but validated with other indicators (i.e. prevalence or serology).

Because of the strong effect of seasonality on the relationships between malaria indicators, it follows that obtaining accurate estimates of transmission across a range of seasonal patterns, not just transmission intensities, is critical for tailoring malaria control and elimination programs to specific country contexts. An accurate map describing seasonal patterns of transmission to attach to maps of transmission intensity and other indicators would be a useful tool. While obtaining this information may not be straightforward, there is a need for research studies designed with measuring not only transmission but also other malaria indicators to ensure the annual pattern of transmission is accounted for. Therefore, goals for reduction in transmission and burden of disease can be further tailored to specific sites.

The methods described here will be able to be compiled into a lookup tool that will allow malaria control professionals to enter the data they have on one index and see the range of likely results for other measures of malaria. In addition to estimates, an essential requirement would be providing a means to display the uncertainty of simulation results. Examples of how this might be achieved are discussed in **Text S3** and shown in **Figures S2–S5 in Text S3**. Such a tool could aid in the planning process of tailoring malaria control interventions to the appropriate level of transmission.

## Supporting Information

Table S1 Datasets used for model validation and their relationship to fitting of OpenMalaria parameters.
(DOCX)

Text S1 Experiment creation and the relationship between parasite prevalence and uncomplicated episodes. This file contains **Table S2** and **Figure S1**.
(DOCX)

Text S2 Methods and results of fitted regression models for the relationships between malaria indicators. This file contains **Tables S3–S4**.
(DOCX)

Text S3 Model choice and presentation of simulation results. This file contains **Figures S2–S5**.
(DOCX)
