# Supplementary material for: Seasonally Dependent Relationships between Indicators of Malaria Transmission and Disease Provided by Mathematical Model Simulations
Source: PLoS Comput Biol. 2014 Sep 4;10(9):e1003812. doi: 10.1371/journal.pcbi.1003812 (PMC4154642; doi:10.1371/journal.pcbi.1003812)
Supplement: Table S1 — Datasets used for model validation and their relationship to fitting of OpenMalaria parameters. (DOCX) [file pcbi.1003812.s001.docx]

**Table S1.** **Datasets used for model validation and their relationship to fitting of OpenMalaria parameters**

| **Source** | **Relationship** | **Dataset description** | **Relationship to model fitting** |
| --- | --- | --- | --- |
| Beier et al. 1999 [[1](#_ENREF_1)] | EIR and parasite prevalence | Observed prevalence in children less than five years of age in 31 sites across Africa. EIR estimated by mosquito capture. | One site as published separately [[2](#_ENREF_2)] was used to fit the model for incidence of asexual blood stage infection [[3](#_ENREF_3)]. |
| Korenromp et al. 2003 [[4](#_ENREF_4)] | Mortality and parasite prevalence | Malaria-specific and all-cause mortality rates as reported by verbal autopsy in children under five in 28 sites across Africa. Parasite prevalence among children under five years in the catchment population of the hospital. | Nine sites (for which EIR estimates were available) were used to fit the model of direct malaria mortality in relation to EIR [[5](#_ENREF_5)]. |
| Okiro et al. 2009 [[6](#_ENREF_6)] | Severe disease and parasite prevalence | Community derived parasite prevalence and the age and clinical presentation of paediatric malaria in children aged 0–9 years admitted to hospital in 13 hospitals across Africa. | None |
| Carneiro et al. 2010 [[7](#_ENREF_7)] | Age-prevalence curves in patterns of differing seasonality | Systematic review of age distribution in children under 10 for clinical malaria, hospital admissions with malaria, and malaria-diagnosed mortality, stratified by level and pattern of transmission. | Some datasets were used for model fitting, but not explicitly considering seasonality of transmission. |

**References**

1. Beier JC, Killeen GF, Githure JI (1999) Short report: entomologic inoculation rates and Plasmodium falciparum malaria prevalence in Africa. Am J Trop Med Hyg 61: 109-113.

2. Beier JC, Oster CN, Onyango FK, Bales JD, Sherwood JA, et al. (1994) Plasmodium falciparum incidence relative to entomologic inoculation rates at a site proposed for testing malaria vaccines in western Kenya. The American journal of tropical medicine and hygiene 50: 529-536.

3. Maire N, Smith T, Ross A, Owusu-Agyei S, Dietz K, et al. (2006) A model for natural immunity to asexual blood stages of Plasmodium falciparum malaria in endemic areas. Am J Trop Med Hyg 75: 19-31.

4. Korenromp EL, Williams BG, Gouws E, Dye C, Snow RW (2003) Measurement of trends in childhood malaria mortality in Africa: an assessment of progress toward targets based on verbal autopsy. Lancet Infect Dis 3: 349-358.

5. Ross A, Maire N, Molineaux L, Smith T (2006) An epidemiologic model of severe morbidity and mortality caused by Plasmodium falciparum. Am J Trop Med Hyg 75: 63-73.

6. Okiro EA, Al-Taiar A, Reyburn H, Idro R, Berkley JA, et al. (2009) Age patterns of severe paediatric malaria and their relationship to Plasmodium falciparum transmission intensity. Malar J 8: 4.

7. Carneiro I, Roca-Feltrer A, Griffin JT, Smith L, Tanner M, et al. (2010) Age-patterns of malaria vary with severity, transmission intensity and seasonality in sub-Saharan Africa: a systematic review and pooled analysis. PLoS One 5: e8988.
