# Supplementary material for: Seasonally Dependent Relationships between Indicators of Malaria Transmission and Disease Provided by Mathematical Model Simulations
Source: PLoS Comput Biol. 2014 Sep 4;10(9):e1003812. doi: 10.1371/journal.pcbi.1003812 (PMC4154642; doi:10.1371/journal.pcbi.1003812)
Supplement: Text S1 — Experiment creation and the relationship between parasite prevalence and uncomplicated episodes. This file contains Table S2 and Figure S1. (DOCX) [file pcbi.1003812.s002.docx]

**Text S1. Experiment creation**

**Seasonality index**

In the OpenMalaria transmission model, daily EIR at each time point EIR*_d_*(*t*) based on a given annual average EIR is specified by five Fourier coefficients representing the cycle’s average (a_0_), annual cycle (a_1_, b_1_), and bi-annual cycle (a_2_, b_2_) as described by:

*$EIR_{d}(t)=e^{a_{0}+a_{1}\cos(\omega t)+b_{1}\sin(\omega t)+a_{2}\cos(2\omega t)+b_{2}\sin(2\omega t)}$,*

where:

$\omega=\frac{2\pi}{T}$,

and T=1 year.

The values a_1_, b_1,_ a_2_, b_2_ are picked to provide a given seasonal profile as described in **Table S1.** For a given annual average EIR, EIR_a_, a_0_ is

$$a_{0}=\ln(\frac{EIR_{a}}{\sum_{t=1}^{T} e^{a_{1}\cos(\omega t)+b_{1}\sin(\omega t)+a_{2}\cos(2\omega t)+b_{2}\sin(2\omega t)}})$$

Parameterization of models of seasonality

1. For simulations of no seasonality in transmission of malaria, values of a_1_=a_2_=b_1_=b_2_=0 were assigned. Simulations were run for the following values of annual average EIR: [0.5, 1, 1.5, 2.5, 4.1, 6.7, 11, 18.2, 30, 49.4, 81.4, 134.3, 221.4, 365].
2. For each annual average EIR, simulations were run for each of six patterns of seasonality (**Table S1, Table 2 of the main manuscript**). Each of these was parameterized with a vector of Fourier coefficients calculated to give the chosen value ϕ [[1](#_ENREF_1),[2](#_ENREF_2)]. The six patterns were selected so as to cover the range of seasonality patterns observed in malaria-endemic areas.

| **Table S2** |  | | | | | |
| --- | --- | --- | --- | --- | --- | --- |
| **Seasonality pattern ID** | **0** | **1,1** | **2,1** | **1,2** | **0.5,2** | **2,2** |
| **Seasonality index (ϕ)** | 0 | 1 | 2 | 1 | 0.5 | 2 |
| **Number of peaks** | 0 | 1 | 1 | 2 | 2 | 2 |
| **a_1_** | 0 | 1.76256 | 4.10688 | 0.836862 | 0.437636 | 2.05344 |
| **a_2_** | 0 | 0 | 0 | 0.836862 | 0.437636 | 2.05344 |
| **b_1_** | 0 | 0 | 0 | 0 | 0 | 0 |
| **b_2_** | 0 | 0 | 0 | 0 | 0 | 0 |
|  |  |  |  |  |  |  |

**Figure S1. Relationship between parasite prevalence and uncomplicated episodes.** Simulated annual pattern of parasite prevalence (unbroken line) and uncomplicated episodes (dashed line) for the seasonality pattern $\phi$ =2, 2 peaks and an annual average EIR of 11. Lines represent the mean over all model variants and multiple random seeds.

**
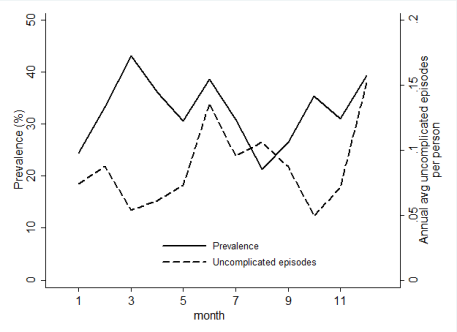
**

**References**

1. Chitnis N, Hardy D, Smith T (2012) A Periodically-Forced Mathematical Model for the Seasonal Dynamics of Malaria in Mosquitoes. Bulletin of mathematical biology 74(5): 1098–1124.

2. Bracewell RN (2000) The Fourier transform and its applicaitons: McGraw-Hill Higher Education. 258-285 p.
