# Supplementary material for: Seasonally Dependent Relationships between Indicators of Malaria Transmission and Disease Provided by Mathematical Model Simulations
Source: PLoS Comput Biol. 2014 Sep 4;10(9):e1003812. doi: 10.1371/journal.pcbi.1003812 (PMC4154642; doi:10.1371/journal.pcbi.1003812)
Supplement: Text S2 — Methods and results of fitted regression models for the relationships between malaria indicators. This file contains Tables S3–S4. (DOCX) [file pcbi.1003812.s003.docx]

**Text S2: Methods fitted regression models for the relationships between malaria indicators**

Statistical model design and fit

The relationships between malaria indicators were investigated by regression analysis using Stata v12 (College Station, Texas). Linear regression models were used for each relationship, based on better fit than Poisson or negative binomial models (as assessed using the Akaike information criteria (AIC)). Final models were fitted using the second-degree fractional polynomial method described in Royston et al. [[1](#_ENREF_1)] and Sauerbrei et al. [[2](#_ENREF_2)] where models are of the form:

$y=\beta_{0}+\beta_{1}x^{p}+\beta_{2}x^{q}$ ,

where *x* and *y* are the two malaria indicators being investigated, transformed as indicated in Table 1 of the manuscript and *p* and *q* are any of (-2, -1, -0.5, 0, 0.5, 1, 2, 3) with *p* ≠ *q* and *x*^0^ representing ln(*x*).

In the case *q=p*:

$y=\beta_{0}+\beta_{1}x^{p}+\beta_{2}x^{p}\ln(x)$.

**Table S3. Fitted regression models for the relationships between malaria indicators, ϕ =0**

| **Variable to estimate*** | **Input variable** |  | **Form** | **Coefficient** | **95% CI** |
| --- | --- | --- | --- | --- | --- |
| EIR | Parasite prevalence | ẞ_1_ | (X+2.68)^3^-9.07 | 0.41 | 0.39,0.44 |
|  |  | ẞ_2_ | (X+2.68)^3^*ln(X+2.68)-6.67 | -0.24 | -0.26,-0.22 |
|  |  | ẞ_0_ |  | 1.65 |  |
|  | Mortality | ẞ_1_ | ln(X)-.91 | -14.70 | -16.76,-12.65 |
|  |  | ẞ_2_ | X^0.5^-1.58 | 27.27 | 24.47,30.06 |
|  |  | ẞ_0_ |  | 1.75 |  |
|  |  |  |  |  |  |
| Parasite prevalence | EIR | ẞ_1_ | (X+0.71)-2.61 | 1.21 | 1.18,1.24 |
|  |  | ẞ_2_ | (X+0.71)^2^-6.83 | -0.13 | -0.13,-0.12 |
|  |  | ẞ_0_ |  | -0.28 |  |
|  | Mortality | ẞ_1_ | X^3^-15.56 | 0.44 | 0.42,0.46 |
|  |  | ẞ_2_ | X^3^*ln(X)-14.23 | -0.28 | -0.30,-0.27 |
|  |  | ẞ_0_ |  | -0.47 |  |
|  |  |  |  |  |  |
| Uncomplicated episodes | EIR | ẞ_1_ | (X+0.71)-2.61 | 0.79 | 0.77,0.82 |
|  |  | ẞ_2_ | (X+0.71)*ln(X+0.71)-2.51 | -0.39 | -0.40,-0.37 |
|  |  | ẞ_0_ |  | 0.43 |  |
|  | Parasite prevalence | ẞ_1_ | (X+2.68)-2.09 | 0.92 | 0.87,0.97 |
|  |  | ẞ_2_ | (X+2.68)^2^-4.35 | -0.19 | -0.20,-0.18 |
|  |  | ẞ_0_ |  | 0.37 |  |
|  | Severe episodes | ẞ_1_ | X^3^-20.02 | 0.32 | 0.25,0.38 |
|  |  | ẞ_2_ | X^3^*ln(X)-20.00 | -0.21 | -0.26,-0.16 |
|  |  | ẞ_0_ |  | 0.24 |  |
|  | Mortality | ẞ_1_ | X^2^-6.23 | 0.96 | 0.91,1.01 |
|  |  | ẞ_2_ | X^2^*ln(X)-5.70 | -0.64 | -0.68,-0.61 |
|  |  | ẞ_0_ |  | 0.41 |  |
|  |  |  |  |  |  |
| Severe episodes | EIR | ẞ_1_ | (X+0.71)^0.5^-1.62 | 0.34 | 0.30,0.38 |
|  |  | ẞ_2_ | (X+0.71)^3^-17.85 | -0.004 | -.004,-.003 |
|  |  | ẞ_0_ |  | 2.83 |  |
|  | Parasite prevalence | ẞ_1_ | (X+2.68)-2.09 | 0.28 | 0.22,0.35 |
|  |  | ẞ_2_ | (X+2.68)^2^-4.35 | -0.05 | -0.07,-0.03 |
|  |  | ẞ_0_ |  | 2.76 |  |
|  | Uncomplicated episodes | ẞ_1_ | (X+0.76)^3^-0.89 | 0.38 | 0.33,0.42 |
|  |  | ẞ_2_ | (X+0.76)^3^*ln(X+0.76)+0.03 | -0.59 | -0.71,-0.47 |
|  |  | ẞ_0_ |  | 2.72 |  |
|  | Mortality | ẞ_1_ | ln(X)-.91 | 3.94 | 3.16,4.72 |
|  |  | ẞ_2_ | X^0.5^-1.58 | -4.86 | -5.92,-3.79 |
|  |  | ẞ_0_ |  | 2.79 |  |
|  |  |  |  |  |  |
| Mortality | EIR | ẞ_1_ | (X+0.71)-2.61 | 0.58 | 0.56,0.59 |
|  |  | ẞ_2_ | (X+0.71)^3^-17.85 | -0.01 | -0.01,-.009 |
|  |  | ẞ_0_ |  | 2.67 |  |
|  | Parasite prevalence | ẞ_1_ | (X+2.68)^2^-4.35 | 0.43 | 0.41,0.46 |
|  |  | ẞ_2_ | (X+2.68)^2^*ln(X+2.68)-3.20 | -0.22 | -0.24,-0.21 |
|  |  | ẞ_0_ |  | 2.52 |  |

*Variables are defined and transformed as per **Table 1** of the main manuscript.

**Table S4. Fitted regression models for the relationships between malaria indicators, ϕ =2, 2 peaks**

| **Variable to estimate*** | **Input variable** |  | **Form** | **Coefficient** | **95% CI** |
| --- | --- | --- | --- | --- | --- |
| EIR | Parasite prevalence | ẞ_1_ | (X+2.84)^3^-6.351870522 | 0.60 | 0.57,0.63 |
|  |  | ẞ_2_ | (X+2.84)^3^*ln(X+2.84)-3.91 | -0.40 | -0.42,-0.37 |
|  |  | ẞ_0_ |  | 1.77 |  |
|  | Mortality | ẞ_1_ | X^-0.5^-.66 | 64.17 | 57.09,71.26 |
|  |  | ẞ_2_ | ln(X)-.84 | 28.63 | 26.13,31.14 |
|  |  | ẞ_0_ |  | 1.78 |  |
|  |  |  |  |  |  |
| Parasite prevalence | EIR | ẞ_1_ | (X+0.71)-2.612658873 | 0.925 | 0.89,0.96 |
|  |  | ẞ_2_ | (X+0.71)^2^-6.83 | -0.08 | -0.09,-.07 |
|  |  | ẞ_0_ |  | -0.79 |  |
|  | Mortality | ẞ_1_ | X^3^-12.59 | 0.47 | 0.43,0.50 |
|  |  | ẞ_2_ | X^3^*ln(X)-10.64 | -0.31 | -0.33,-0.28 |
|  |  | ẞ_0_ |  | -0.92 |  |
|  |  |  |  |  |  |
| Uncomplicated episodes | EIR | ẞ_1_ | (X+0.71)-2.61 | 0.70 | 0.67,0.73 |
|  |  | ẞ_2_ | X*ln(X+0.71)-2.51 | -0.32 | -0.33,-0.30 |
|  |  | ẞ_0_ |  | 0.26 |  |
|  | Parasite prevalence | ẞ_1_ | (X+2.84)-1.85 | 0.76 | 0.73,0.79 |
|  |  | ẞ_2_ | (X+2.84)^3^-6.35 | -0.05 | -0.05,-0.05 |
|  |  | ẞ_0_ |  | 0.26 |  |
|  | Severe episodes | ẞ_1_ | X^3^-18.30 | 0.33 | 0.26,0.41 |
|  |  | ẞ_2_ | X^3^*ln(X)-17.74 | -0.24 | -0.29,-0.18 |
|  |  | ẞ_0_ |  | 0.11 |  |
|  | Mortality | ẞ_1_ | X-2.33 | 2.76 | 2.54,2.98 |
|  |  | ẞ_2_ | X^2^-5.41 | -0.52 | -0.57,-0.47 |
|  |  | ẞ_0_ |  | 0.20 |  |
|  |  |  |  |  |  |
| Severe episodes | EIR | ẞ_1_ | (X+0.71)-2.61 | 0.33 | 0.29,0.38 |
|  |  | ẞ_2_ | (X+0.71)*ln(X)-2.51 | -0.18 | -0.20,-0.15 |
|  |  | ẞ_0_ |  | 2.74 |  |
|  | Parasite prevalence | ẞ_1_ | (X+2.84)^2^-3.43 | 0.20 | 0.17,0.23 |
|  |  | ẞ_2_ | (X+2.84)^2^*ln(X+2.84)-2.11 | -0.17 | -0.19-0.14 |
|  |  | ẞ_0_ |  | 2.73 |  |
|  | Uncomplicated episodes | ẞ_1_ | ln(X+0.81)+0.13 | 0.08 | 0.06,0.11 |
|  |  | ẞ_2_ | (X+0.81)^2^-0.78 | 0.14 | 0.10,0.18 |
|  |  | ẞ_0_ |  | 2.64 |  |
|  | Mortality | ẞ_1_ | X^0.5^-1.53 | 9.36 | 7.30,11.41 |
|  |  | ẞ_2_ | X^0.5^*ln(X)-1.29 | -3.24 | -3.99,-2.49 |
|  |  | ẞ_0_ |  | 2.70 |  |
|  |  |  |  |  |  |
| Mortality | EIR | ẞ_1_ | (X+0.71)-2.61 | 0.57 | 0.55,0.59 |
|  |  | ẞ_2_ | (X+0.71)^2^-6.83 | -0.05 | -0.06,-0.05 |
|  |  | ẞ_0_ |  | 2.46 |  |
|  | Parasite prevalence | ẞ_1_ | (X+2.84)^2^-3.43 | 0.48 | 0.46,0.51 |
|  |  | ẞ_2_ | (X+2.84)^2^*ln(X+2.84)-2.11 | -0.29 | -0.31,-0.27 |
|  |  | ẞ_0_ |  | 2.39 |  |

*Variables are defined and transformed as per **Table 1** of the main manuscript.

**References**

1. Royston P, Altman DG (1994) Regression Using Fractional Polynomials of Continuous Covariates: Parsimonious Parametric Modelling. Journal of the Royal Statistical Society Series C (Applied Statistics) 43: 429-467.

2. Sauerbrei W, Roston P (1999) Building multivariable prognostic and diagnostic models: transformation of the predictors by using fractional polynomials. Journal of the Royal Statistical Society Series A 162: 71-94.
