# Supplementary material for: Seasonally Dependent Relationships between Indicators of Malaria Transmission and Disease Provided by Mathematical Model Simulations
Source: PLoS Comput Biol. 2014 Sep 4;10(9):e1003812. doi: 10.1371/journal.pcbi.1003812 (PMC4154642; doi:10.1371/journal.pcbi.1003812)
Supplement: Text S3 — Model choice and presentation of simulation results. This file contains Figures S2–S5. (DOCX) [file pcbi.1003812.s004.docx]

**Text S3. Model choice and presentation of simulation results**

When presenting results of stochastic simulation models, it is important not only to demonstrate the general trends, but to adequately express uncertainty inherent in stochastic simulations. Evaluating goodness of fit and uncertainty for simulation results of model ensembles remains a challenge [[1](#_ENREF_1)], as does presentation of these results. There are numerous options for analysing and presenting simulation results, including aggregation into averages, ranges and standard deviations, elimination of poor-performing models, and weighting models based on their structure similarity or performance during the fitting process. The following examples represent analysis of simulation results from the OpenMalaria model ensemble of 14 model variants as described in Smith et. al [[2](#_ENREF_2)].

In the context of this study the options relevant options include:

1. As presented in the main manuscript, scatter plot range of results with the fitted regression model over all model variants of each pattern of seasonality (**Figure S2**)
2. Shaded range of results with the median over all model variants of each pattern of seasonality (**Figure S3**)
3. Shaded range of results with the median over all seeds for each model variant, for each pattern of seasonality (**Figure S4**)
4. Shading range of results with the mean of all model variants and the fitted regression model over all model variants, for each pattern of seasonality (**Figure S5**)

There are benefits to displaying results by model variant if the goal of analysis is to understand the effect differences in assumptions of model structure have on results. For example, model variants 670, 674 and 678 have different assumptions about susceptibility to co-morbidity and access to treatment [[2](#_ENREF_2)], which may be of interest when examining the relationship between malaria mortality and EIR (**Figure S4**). However, if model variants are not to be examined individually, there are benefits of displaying results of means or medians across all model variants as the overall uncertainty is more relevant to analysis of results than the uncertainty due to any one model variant.

While a shaded area gives an easily-identifiable range of simulation results encompassing areas not explicitly simulated in the experiment, (**Figure S3**), a scatter plot is able to identify outliers in directions of both independent and dependent variables as well as give an indication of the density of simulation results in a given area (**Figure S2**), more easily showing patterns in the overall results and how they relate to the summary smoothing functions.

In the case of the relationship between mortality and EIR, the uncertainty in simulation results is due more to model variant than to pattern of seasonality, as can be ascertained by comparing **Figure S3** and **Figure S4**. However, this is not the case for all relationships between indicators presented in this study.

Fractional polynomial regression has the flexibility to fit the range of non-linear, non-monotonic relationships seen between these indicators and has the advantage of being able to exclude predictions less than zero through the algorithms used by defining the origin. Despite criticisms about the fractional polynomial approach to model selection and potential inflation of type one error [[3](#_ENREF_3)], choosing to present results of regression analysis allows a methodology for applying these equations to make predictions in areas outside the range of simulations, which means and medians of simulation results cannot. The fitted models will diverge from resembling the means for relationships that are increasingly non-linear.

**Figure S2. Fitted regression models for each pattern of seasonality over scatter range of simulation results**

Circles represent simulated results of the relationship between mortality and eir over 14 model variants, six random seeds and five patterns of seasonality. Lines represent, for each pattern of seasonality as described in **Figure 1** and **Table 2**, the fractional polynomial regression model fitted over 14 model variants as described in Smith et. al [[2](#_ENREF_2)] and five random seeds. Unbroken red line represents φ =0. Brown dashed line represents φ =1, one peak. Orange dotted-dashed line represents φ =1, two peaks. Green dotted line represents φ =2, one peak. Black dotted-dashed line represents φ =2, two peaks. Blue dashed line represents φ =0.5, two peaks.

**
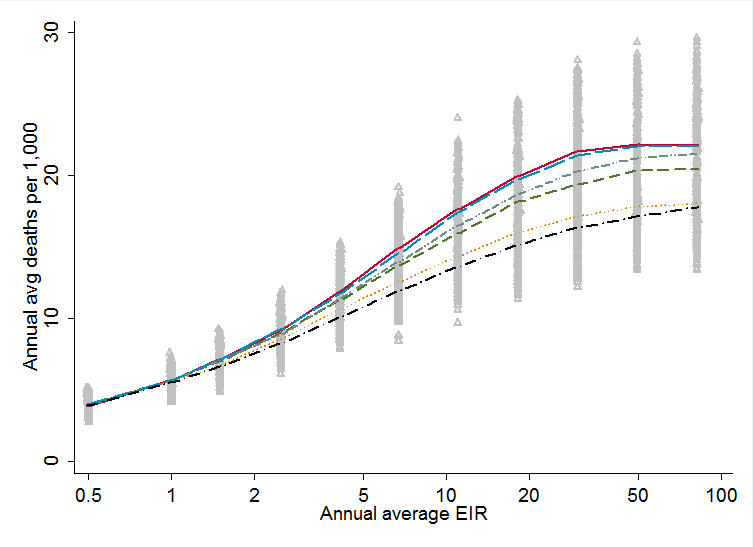
**

**Figure S3. Median of each pattern of seasonality over shaded range of simulation results**

Shaded area represents range of simulated results of the relationship between mortality and EIR over 14 model variants, five random seeds and six patterns of seasonality. Lines represent, for each pattern of seasonality as described in **Figure 1** and **Table 2**, the median of 14 model variants as described in Smith et. al [[2](#_ENREF_2)] and five random seeds. Unbroken red line represents φ =0. Brown dashed line represents φ =1, one peak. Orange dotted-dashed line represents φ =1, two peaks. Green dotted line represents φ =2, one peak. Black dotted-dashed line represents φ =2, two peaks. Blue dashed line represents φ =0.5, two peaks.


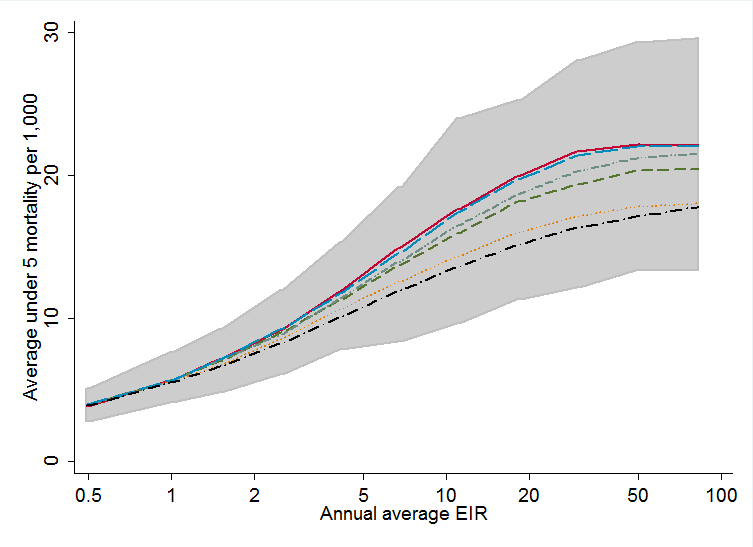


**Figure S4. Median of each model variant over shaded range of simulation results, by pattern of seasonality**

Shaded areas represent the range of simulated results of the relationship between mortality and eir over 14 model variants and five random seeds. Lines represent the median of each of the 14 model variants as described in Smith et. al [[2](#_ENREF_2)] over five random seeds. Panels represent the patterns of seasonality as described in **Figure 1** and **Table 2**.


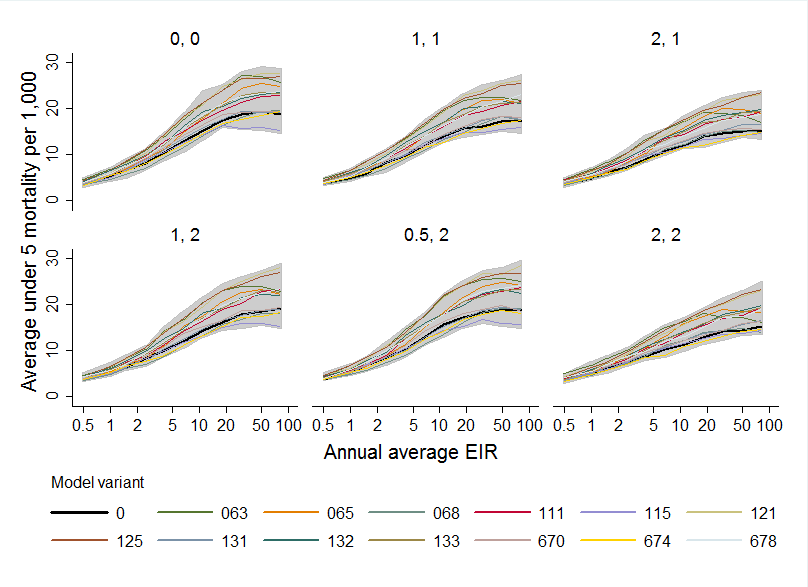


**Figure S5. Mean and fitted regression model over shaded range of simulation results, by pattern of seasonality**

Shaded areas represent the range of simulated results of the relationship between mortality and EIR over 14 model variants and five random seeds. Black lines represent the mean of 14 model variants as described in Smith et. al [[2](#_ENREF_2)] and five random seeds. Green lines represent the fractional polynomial regression model fitted over 14 model variants and five random seeds. Panels represent the patterns of seasonality as described in **Figure 1** and **Table 2**.


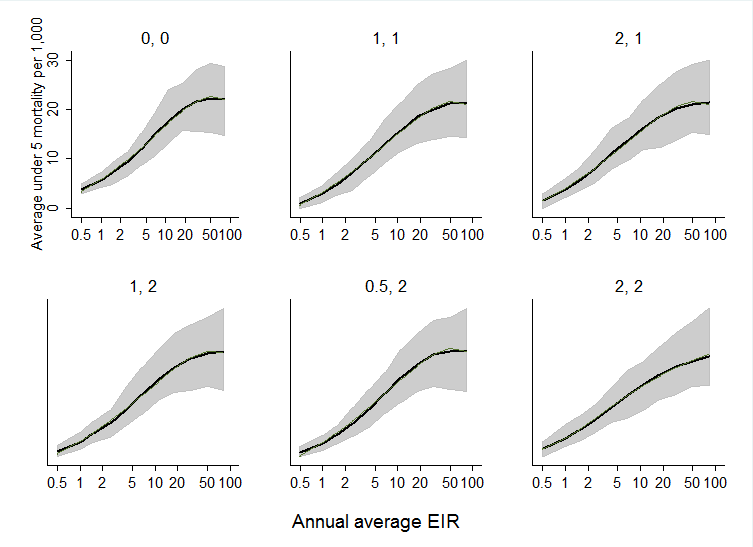


**References**

1. Knutti R (2010) The end of model democracy? An editorial comment. Climactic Change 102: 395-404.

2. Smith T, Ross A, Maire N, Chitnis N, Studer A, et al. (2012) Ensemble modeling of the likely public health impact of a pre-erythrocytic malaria vaccine. PLoS medicine 9: e1001157.

3. Strasak AM, Umlauf N, Pfeiffer RM, Lang S (2011) Comparing penalized splines and fractional polynomials for flexible modelling of the effects of continuous predictor variables. Computational Statistics & Data Analysis 55: 1540-1551.
